# Mucosal microbiota characterization in gastric cancer identifies immune-activated–related transcripts relevant gastric microbiome signatures

**DOI:** 10.3389/fimmu.2024.1435334

**Published:** 2024-09-23

**Authors:** Chengjia Qian, Jiang Hui, Ziyao Peng, Xiaoyan Sun, Jiali Zhang

**Affiliations:** ^1^ Department of Gastrointestinal Surgery, Affiliated Hospital of Jiangnan University, Wuxi, China; ^2^ Department of Trauma-Emergency & Critical Care Medicine, Shanghai Fifth People’s Hospital, Fudan University, Shanghai, China; ^3^ Department of Nuclear Medicine, Shanghai Fifth People’s Hospital, Fudan University, Shanghai, China; ^4^ Central Laboratory, Shanghai Fifth People’s Hospital, Fudan University, Shanghai, China

**Keywords:** mucosal microbiota, gastric cancer, immune-activated, transcripts, gastric microbiome signatures

## Abstract

Tumor microenvironment (TME) immune cells and gastric mucosal microbiome constitute two vital elements of tumor tissue. Increasing evidence has elucidated their clinicopathological significance in predicting outcomes and therapeutic efficacy. However, comprehensive characterization of immune cell-associated microbiome signatures in the TME is still in the early stages of development. Here, we characterized the gastric mucosa microbiome and its associations with immune-activated related transcripts (IATs) in 170 GC tumor tissues and matched non-tumor tissues using 16s rRNA gene sequencing and quantitative reverse transcription-PCR. Microbial diversity and richness were significantly higher in GC tumor tissues than in non-tumor tissues. Differences in microbial composition between the groups were evident, with Firmicutes, Proteobacteria, Bacteroidota, Campilobacterota, Actinobacteria, Fusobacteriota, Verrucomicrobiota, Acidobacteriota, and Cyanobacteria being the dominant phyla in the gastric mucosal microbiota. Microbial interaction network analysis revealed distinctive centralities of oral bacteria (such as *Fusobacterium, Porphyromonas, Prevotella*, etc.) in both tumor and normal mucosae networks, suggesting their significant influence on GC microbial ecology. Furthermore, we analyzed the expression of IATs (CXCL9, CXCL10, GZMA, GZMB, PRF1, CD8A, IFNG, TBX2, and TNF) and characterized IAT-relevant gastric microbiome signatures in GC patients. Our results showed that the expression of CXCL9, CXCL10, GZMA, GZMB, PRF1 and IFNG was significantly higher in tumor tissues than in adjacent normal tissues in GC patients. Notably, high expression of IATs in tumor tissues was associated with improved survival in GC patients and could serve as a powerful predictor for disease-free survival. Additionally, analysis of IAT levels and mucosal microbiota diversity revealed a correlation between higher IAT expression and increased microbiota richness and evenness in the IATs ^high^ group, suggesting potential interactions between mucosal microbiota and tumor immunopathology. Spearman correlation analysis showed positive associations between IAT expression and specific mucosal bacterial species. Notably, *Akkermansia muciniphila* demonstrated potential involvement in modulating GZMB expression in the GC mucosal microenvironment. These findings underscore the importance of mucosal microbiota alterations in GC and suggest potential therapeutic targets focusing on modulating the tumor microbiota for improved clinical outcomes. The detailed characterization of these elements has profound implications for both treatment and survival prediction in GC. We observed that microbial diversity and richness were significantly higher in GC tumor tissues compared to non-tumor tissues. These differences highlight the unique microbial landscape of GC tumors and suggest that the microbiome could influence tumor development and progression. Importantly, our study demonstrated that high expression levels of IATs in GC tumor tissues were associated with improved patient survival. This suggests that IATs not only reflect immune activation but also serve as valuable biomarkers for predicting disease-free survival. The potential of IATs as predictive markers underscores their utility in guiding therapeutic strategies and personalizing treatment approaches. Moreover, the correlation between higher IAT expression and increased microbiota richness and evenness suggests that a diverse and balanced microbiome may enhance immune responses and contribute to better clinical outcomes. These findings highlight the critical need to consider mucosal microbiota alterations in GC management. Targeting the tumor microbiota could emerge as a promising therapeutic strategy, potentially leading to more effective treatments and improved patient outcomes. Understanding and modulating the microbiome’s role in GC opens new avenues for innovative therapeutic interventions and personalized medicine.

## Introduction

Gastric cancer (GC) is the fifth most common cancer worldwide and over 1 million new cases were diagnosed in 2020 (1). In China, GC was responsible for more than 509,421 new cases and 400,415 deaths in 2022 (2), making it the third most frequently diagnosed cancer and the third leading cause of cancer-related deaths. One of the primary risk factors for GC is infection with *Helicobacter pylori*, as the majority of GC cases are associated with this pathogen (3). Advances in sequencing technology have revealed that the stomach hosts a diverse microbiota beyond *H. pylori*. Notably, studies have found that the microbiota in GC patients was associated with decreased diversity and richness compared with intestinal metaplasia (4). Understanding how the microbiota composition in *H. pylori*-positive GC patients affects the local tumor microenvironment (TME) warrants further investigation.

To assess the immune contexture within the TME, numerous models (5–7) utilizing immunoscoring have been developed. These models provide robust statistical parameters for prognostic evaluation and therapeutic efficacy across various solid tumors, including GC (8). Traditionally, immunohistochemistry has been the predominant method for investigating cellular heterogeneity. However, immunohistochemistry has limitations, including a restricted set of phenotypic markers and the requirement for sizable biopsy specimens. Technical constraints in turn resulted in studies marked by small sample sizes, a scarcity of cell types, or both. Additionally, achieving standardized and reproducible staining intensity measurement, crucial for accurate protein expression quantification, remains inherently challenging in immunohistochemistry.

Recent innovations in prognostic tools aim to improve survival predictions post-GC diagnosis. These tools employ a novel computational algorithm to enumerate immune cell subsets from RNA specimens sourced from various tissue types, encompassing solid tumors (9–11). Furthermore, contemporary immune profiling studies have delved into the cytokine and chemokine milieu characterizing each gene cluster predictive of survival in patient cohorts sharing identical TNM stages. Analysis of the expression patterns of selected cytokine and chemokine mRNAs in 299 GC samples unveiled CXCL9, CXCL10, GZMA, GZMB, PRF1, CD8A, IFNG, TBX2, and TNF as immune activation-related transcripts (IATs), serving as robust statistical parameters for prognostic assessments in GC patients (10). Notably, CXCL9 and CXCL10 have been shown to cooperate in recruiting effector T cells into tumors. Newly strategies including plasmid-borne CXCL9 (12), intratumor injection of CXCL9 (13), recombinant CXCL10 protein with adoptive cell therapy (ACT) (14), intra-tumor injection of CXCL10 (15), retroviral transduction tumor cells with CXCL10 (16, 17) were effective in increasing T cell infiltration and reducing tumor growth in animal models (18). Despite their potential, these strategies have yet to be explored in clinical trials, partly due to challenges such as the limited bioavailability of injected proteins. Additionally, research has indicated that commensal bacteria colonizing could trigger activation of immune cells to express the chemokine CXCL10 which leaded to the formation of CXCL10–bacterial DNA complexes (19). The gastrointestinal mucosa is a well-studied interface for microbiota-IAT interactions. However, profiling of mucosal microbiota and IATs associated microbiome in the GC patients were lack. Therefore, further studies analyzing these interactions in gastric mucosa from cancer patients are urgently needed.

Recent advances in high-throughput sequencing based on conserved 16S ribosomal RNA and newly developed computational methods have uncovered a complex and distinct bacterial community that inhabits in the tumor mucosa compared with non-tumor mucosa, in addition to *H. pylori*. Species such as *Prevotella melaninogenica, Streptococcus anginosus* and *Propionibacterium acnes* have been identified (20, 21). It remains unclear whether the presence of *H. pylori* shapes the microbiota composition in gastric mucosa compared with non-tumor mucosa. Some studies suggest that *H. pylori* infection induces inflammation in the gastric mucosa, with changes in gastric acid and gastrin secretion, resulting in the gastric mucosa bacterial shifting (22, 23). However, the microbial profiling of GC mucosa with *H. pylori* infection and its association with IATs remain scarce.

To address this gap, we conducted this study employing 16s rRNA gene sequencing on tumor tissues and matched non-tumor tissues from 85 GC patients with *H. pylori* infection. This approach allowed us to characterize the mucosa-associated microbiota comprehensively. We also performed quantitative reverse transcription-PCR analysis of the paired GC tissue samples to quantify key IATs, including CXCL9, CXCL10, GZMA, GZMB, PRF1, CD8A, IFNG, TBX2, and TNF. By combining these analyses, we aimed to identify IATs relevant gastric microbiome signatures.

## Results

### Altered gastric mucosal microbiota in GC tumor tissues compared with matched non-tumor tissues

**Figure 1:**
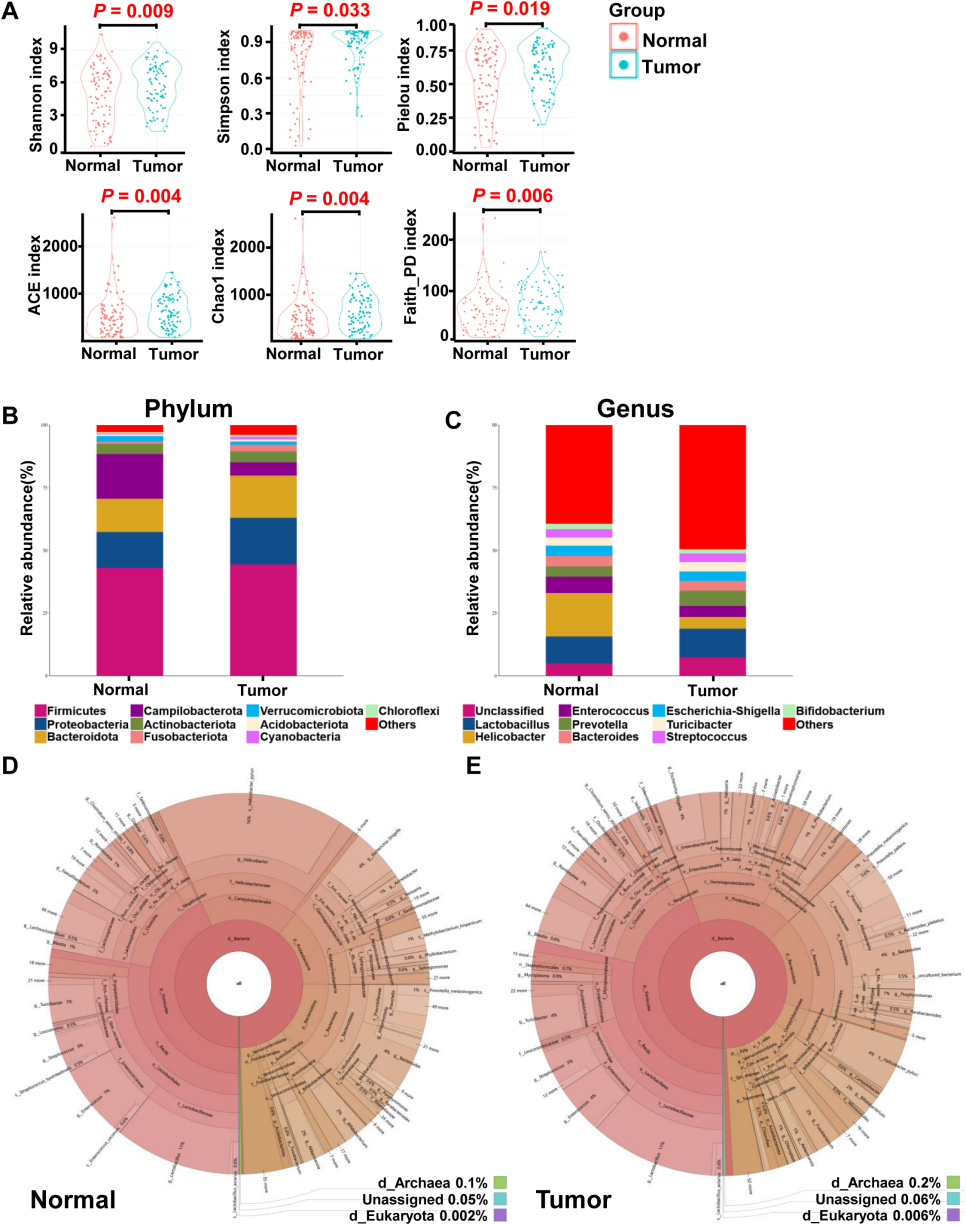
Overall structure and composition map of the gastric microbiota in the tumor and matched normal mucosae tissues. The α-diversity indices (Shannon, Simpson, Pielou, ACE, Choa and Faith_PD) were used to evaluate the overall structure of mucosae microbiota in GC patients **(A)**. The two-sided Wilcoxon signed rank test was utilized to analyze variations between tumor and adjacent normal mucosae. The microbiota structures at phylum **(B)** and genus **(C)** levels in both tumor and normal mucosae tissues are depicted, along with Krona species composition plots for normal **(D)** and tumor **(E)** tissues.

In this study, we investigated the microbial composition of gastric tumor tissues and compared it with matched non-tumor tissues from GC patients (**Table 1**), focusing on alterations in gastric mucosal microbiota. 16s rRNA gene sequencing yielded a median of 73,634 clean reads for 170 paired tumor and non-tumor tissues. To assess differences in microbial diversity, we analyzed alpha diversity measures. The observed OTUs, which reflects species richness, were significantly higher in tumor tissues than in non-tumor tissues (623.68 vs. 493.00; P = 0.01; **Supplementary Figure S1**). Additional alpha diversity indices, such as the Shannon, Simpson, and Pielou indices, also showed higher values in tumor tissues (P = 0.009; P = 0.033; P = 0.019; **Figure 1A**). Similarly, indices measuring species evenness, including ACE, Chao1, and Faith_PD, were significantly higher in tumor tissues (P = 0.004; P = 0.004; P = 0.006; **Figure 1A**). However, due to significant inter-individual variation, principal coordinate analysis (PCoA) could not separate the tumor and non-tumor mucosa microbiomes into distinct clusters (**Supplementary Figure S1**).

**Table 1:** Characteristics of Patients.

Characteristics	Patients (n = 85)
Age (means ± SD)	65.44± 21.56
Gender (Female/Male)	30/55
Weight (Kg, means ± SD)	66.1 ± 25.9
Height (cm, means ± SD)	166.5± 16.5
BMI (means ± SD)	23.73± 5.67
Complications, no	
Hypertension	35
Diabetes mellitus	10
Tumor localization, no	
Proximal stomach	21
Body/Fundus	25
Antrum	39
Tumor differentiation, no	
High differentiated	2
Moderately/poor differentiated	83
Lauren typing, no	
Intestinal type	16
Diffuse type	8
Mixed type	61
Tumor stage, no	
I (Ia, Ib)	12
II (IIa, IIb)	19
III (IIIa, IIIb, IIIc)	46
IV	8
HP infection,	
Positive	85
Negative	0
Antibiotics use, no	0
Pre-operative chemotherapy, no	0

BMI, Body mass index; HP, Helicobacter pylori; no, number; SD, standard deviation.

**Figure 2:**
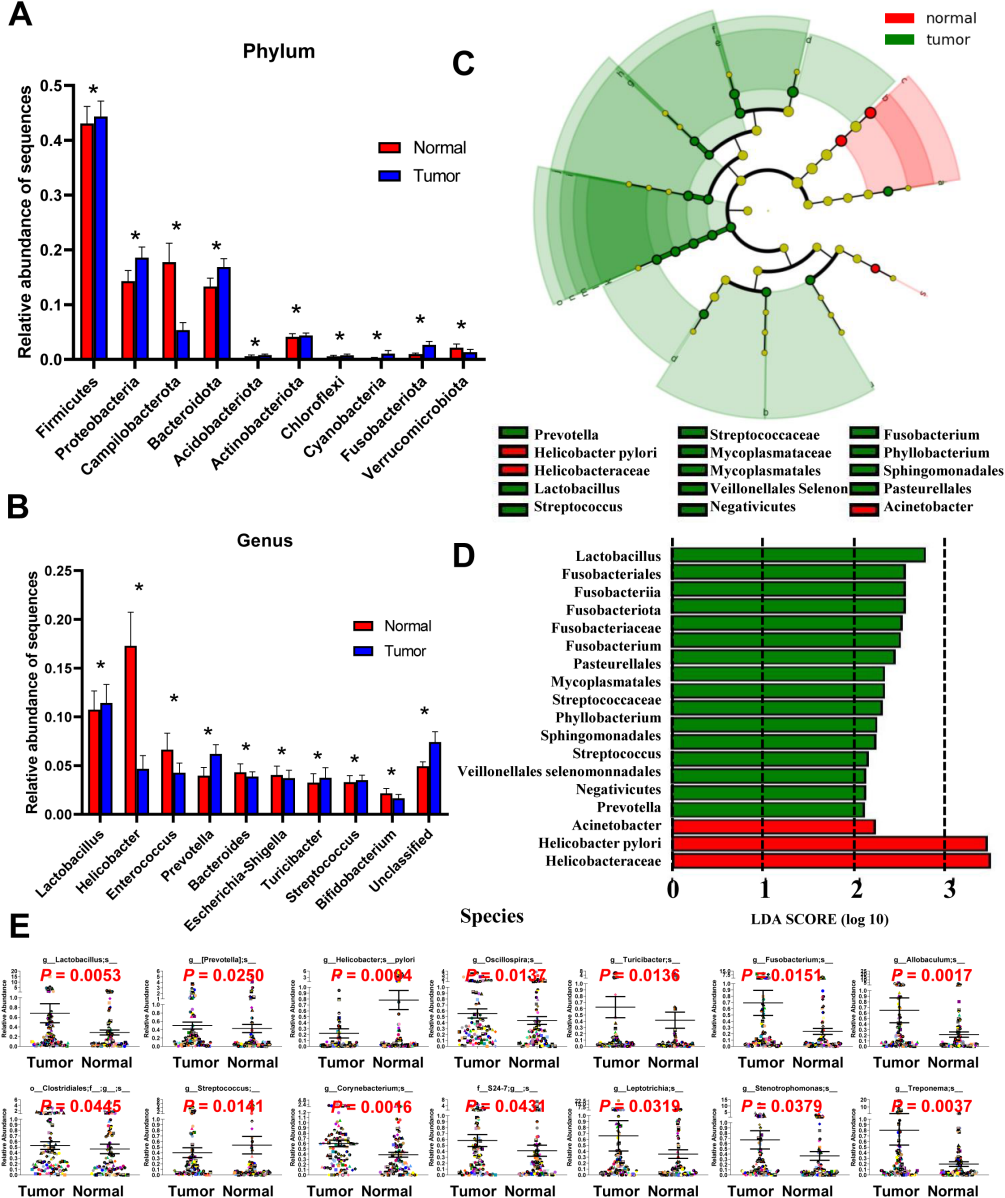
Different bacterial taxa between tumor and matched normal mucosae microbiomes. Relative abundance comparisons at the bacterial phylum **(A)** and genus **(B)** levels are presented; *P<0.05. The LEfSe analysis identifies taxa with significant differences in abundance between tumor and adjacent tissues **(C)**, with only those exceeding a significant LDA threshold value of >2 displayed **(D)**. Fourteen differentially abundant bacterial species were identified **(E)**, and representative dot plots illustrate their relative abundances, showing significant differences between tumor and adjacent tissues.

Given the observed differences in gastric microbiota between non-tumor and tumor tissues, which were dominated by Firmicutes, Proteobacteria, Bacteroidota, Campilobacterota, Actinobacteria, Fusobacteriota, Verrucomicrobiota, Acidobacteriota, and Cyanobacteria (**Figures 1B**, **2A**), we hypothesized that there is a shift in mucosal microbiome profiles in GC patients. The top 10 genera showed in **Figure 1C** included *Lactobacillus*, *Helicobacter*, *Enterococcus*, *Prevotella*, *Bacteroides*, *Escherichia-Shigella*, *Turicibacter*, *Streptococcus*, and *Bifidobacterium*. Notably, the Proteobacteria/Campylobacterales ratio was significantly higher in the tumor mucosa group (p = .000; **Figures 1D, E**).

ANOVA was used to assess mean differences in species abundance at various taxonomic levels. Significant differences were observed at the phylum and genus levels (**Figures 2A**, **B**). At the phylum level, differences were noted inFirmicutes, Proteobacteria, Campilobacterota, Bacteroidota, Acidobacteriota, Actinobacteriota, Chloroflexi, Cyanobacteria, Fusobacteriota, and Verrucomicrobiota. At the genus level, differences were observed in *Lactobacillus*, *Helicobacter*, *Enterococcus*, *Prevotella*, *Bacteroides*, *Escherichia-Shigella*, *Turicibacter*, *Streptococcus*, and *Bifidobacterium*. Discriminant analyses using LEfSe identified 18 bacterial phylotypes that were significantly different between GC tumor and normal mucosa microbiota (**Figures 2C, D**). The tumor microbiomes, present in over 90% of the patients, () exhibited increased abundances of several taxa, including *Lactobacillus*, *Prevotella*, *Clostridiales*, *Oscillospira*, *Turicibacter*, *Fusobacterium*, *Corynebacterium*, *Leptotrichia*, *Stenotrophomonas*, *Allobaculum*, *Treponema* and the family S24-7 (**Figure 2E**). Of note, taxa enriched in the normal mucosa microbiomes included *Helicobacter pylori* and *Streptococcus* genera, consistent with previous reports (19). A heatmap depicting the most abundant genera identified in GC mucosa microbiota showed correlations between the mucosal microbiome and the abundance of selected genera (**Supplementary Figure S2**).

### Significant centralities of oral bacteria in GC mucosae ecological network

**Figure 3:**
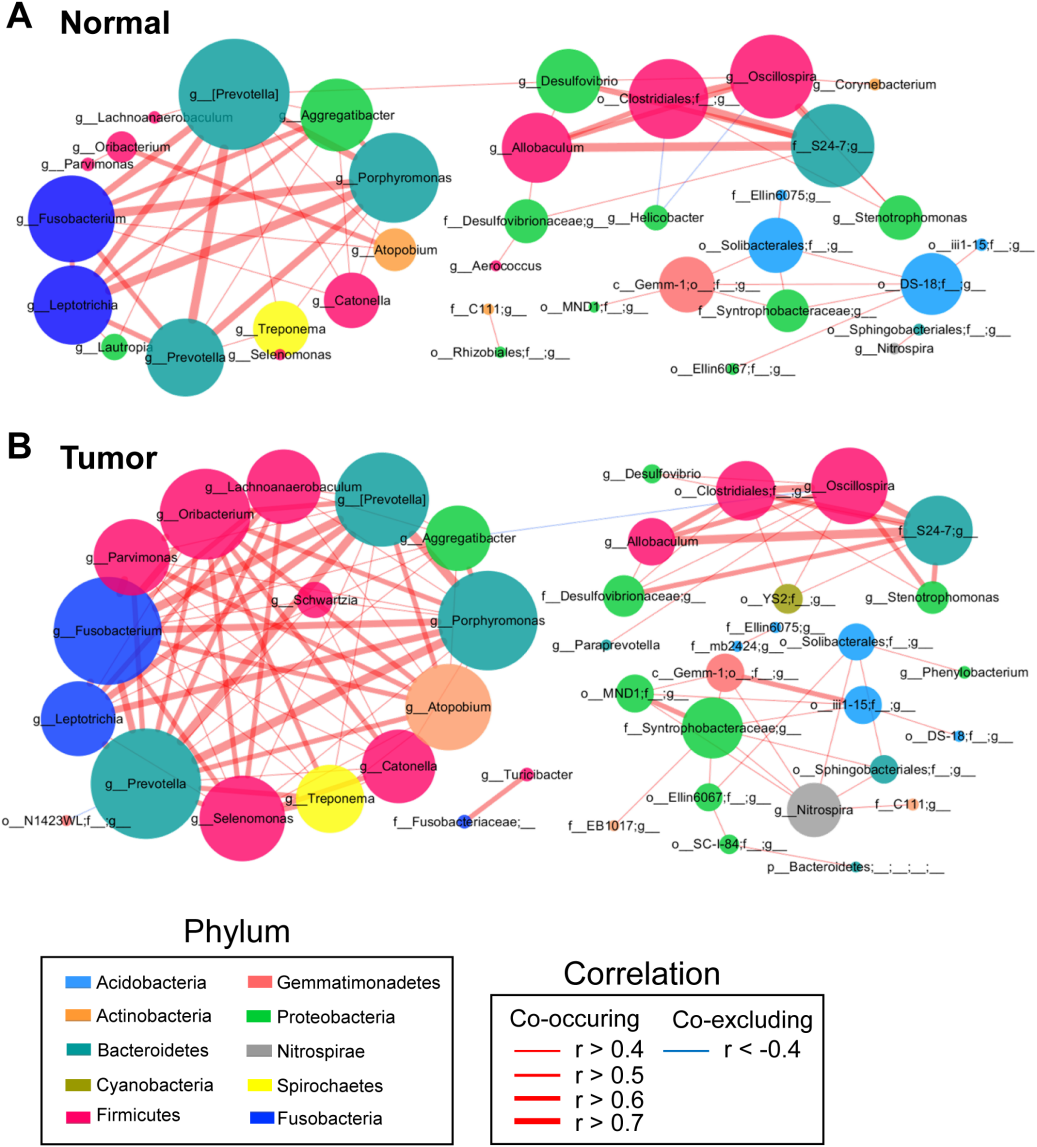
Co-occurrence network analysis of gastric bacterial genera with correlation coefficient >0.4 or < –0.4 in normal mucosae **(A)**, matched tumor mucosae **(B)**. SparCC algorithm was used for correlation coefficient calculation, and Cytoscape version 3.7.0 facilitated network construction. Red and blue lines represent positive and negative correlations, respectively.

SparCC algorithm-generated correlation-based microbial interaction networks identified co-occurrence and co-excluding interactions, highlighting the roles of oral bacteria such as *Fusobacterium*, *Porphyromonas*, *Prevotella*, *Leptotrichia*, *Aggregatibacter*, *Oribacterium*, *Parvimonas*, *Atopobium*, *Treponema*, and *Selenomonas* in both tumor and adjacent normal mucosae networks (**Figures 3A, B**; **Supplementary Figures S4A, B**).

To identify potential biomarkers, we focused on operational taxonomic units (OTUs) with significant weighted node connectivity (WNC) scores. This analysis identified *Prevotella*, *Porphyromonas*, *Fusobacterium*, *Aggregatibacter*, *Parvimonas*, *Oribacterium*, *Leptotrichia*, *Catonella*, *Atopobium*, *Allobaculum*, *Oscillospira*, *Lachnoanaerobaculum*, and *Selenomonas* as significant in the tumor mucosea network (**Figure 3B**). Furthermore, it highlighted differential microbial enrichment patterns between normal and tumor mucosae (**Supplementary Figure S4B**). OTU identification in the normal mucosea network included *Prevotella pallens*, *Eubacterium biforme* and *Helicobacter pylori*, which formed the backbone of the normal mucosae-specific network and likely exerted significant influence on normal microbial ecology (**Supplementary Figure S4A**). Given this, our results could suggest potential cooperation interactions among these species in the microenvironment of GC-associated gastric mucosae.

### Mucosal microbiota richness and diversity is significantly higher in IATs^high^ group in GC tissues

**Figure 4:**
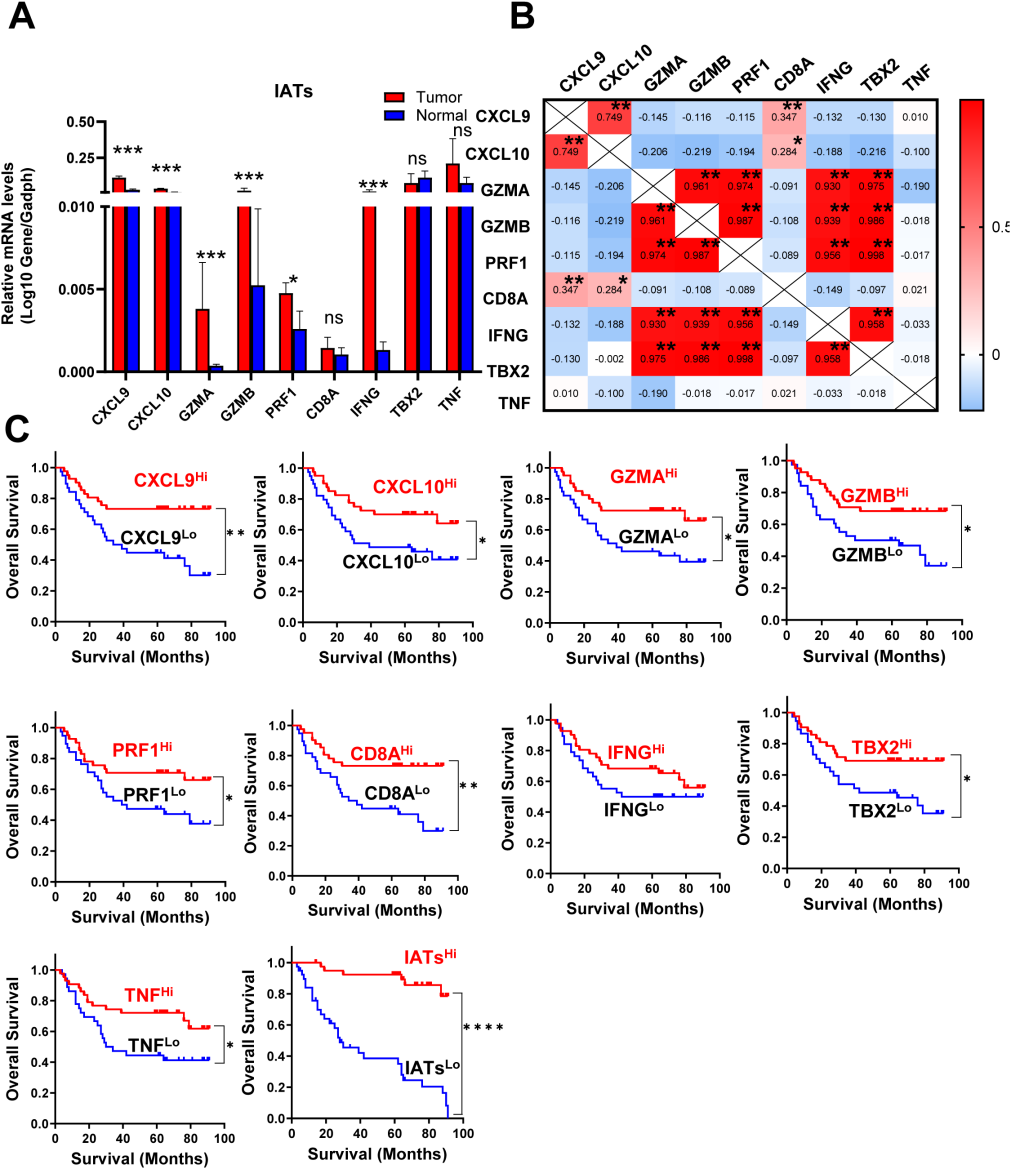
IATs were selectively regulated in tumor and the prognostic significance of IATs in GC patients. **(A)** Quantitative real-time polymeras chain reaction (qRT-PCR) was employed to detect the mRNA expression of each IATs in tumor and adjacent normal tissues (n=85). **(B)** Values denote the Pearson correlation coefficients; values closer to 1 indicate a better correlation. *P<0.05; **P<0.001; ***P<0.0001; ****P<0.00001; ns, no significant difference. **(C)** Cumulative OS times were calculated by the Kaplan-Meier method and analyzed by the log-rank test. The patients were divided into two groups according to the median value of CXCL9, CXCL10, GZMA, GZMB, PRF1, CD8A, IFNG, TBX2, TNF and IATs in tumor tissues.

Given the intrinsic gene expression signature closely linked to stromal activation and immune activation processes, we aimed to determine whether the IATs could accurately predict outcomes. This study focused on mainly IATs, namely CXCL9, CXCL10, GZMA, GZMB, PRF1, CD8A, IFNG, TBX2, and TNF. Quantitative PCR was performed to analyze the mRNA expression of these IATs in tumor and adjacent normal tissues from 85 GC patients. The levels of CXCL9, CXCL10, GZMA, GZMB, PRF1, CD8A, IFNG and TNF were significantly higher in tumor tissues compared to adjacent normal tissues whereas the TBX2 level was significantly lower in tumor tissues (**Figure 4A**). Relevance analyses revealed that several IATs were significantly associated with each other, indicating that different IATs might be co-regulated during tumor progression (**Figure 4B**). Moreover, Kaplan-Meier survival curves were plotted to investigate associations with survival. Positive correlations were observed between the expression levels of CXCL9, CXCL10, GZMA, GZMB, PRF1, CD8A, TNF and IATsand overall survival (OS) in tumor tissues (**Figure 4C** P = 0.0021; P = 0.0264; P = 0.0132; P = 0.0185; P = 0.026; P = 0.002; P = 0.0182; P = 0.0245; P < 0.0001). However, no significant correlation was found for IFNG in tumors or for these chemokines and cytokines in adjacent normal tissues (**Figure 4C**, data not showed). Multivariate Cox proportional hazards analysis was performed, and variables that were associated with survival by univariate analysis were adopted as covariates. In multivariate analysis, the expression level of TNF and IATs in tumor could emerge as an independent prognostic factor of either OS (HR, 0. 217; 95%CI, 0. 088-0. 536; P = 0.001; **Table 2**) or DFS (HR, 0.254; 95%CI, 0.105-0.618; P = 0.003; **Table 2**). These results suggested that IATs were significantly associated with GC progression and could serve as a powerful predictor of GC patient disease-free survival.

**Table 2:** Univariate and Multivariate Analyses of Factors Associated with Survival and Recurrence.

Variables	OS				DFS			
	Univariate *P*	Multivariate			Univariate *P*	Multivariate		
		HR	95% CI	*P*		HR	95% CI	*P*
Age, years (>64/≤64)	.241			NA	.348			NA
Gender (female/male)	.995			NA	. 230			NA
Tumor stage (pT4/pTis+pT1+pT2+pT3)	**.0019**			NA	**.0034**			NA
Nodal status (pN1+pN2/pN0)	**.0017**	17.681	3.753-83.292	**.000**	**.0008**	5.790	2.158-15.534	**<.0001**
Distant metastases (Pos/Neg)	**.0019**	3.429	1.118-10.516	**.031**	**.0005**	3.415	1.410-8.269	**.006**
Differentiation(H+M/L)	.199			NA	.198			NA
CXCL9_high_/CXCL9_low_ tumor	**0.0021**			NA	**0.004**			NA
CXCL10_high_/CXCL10_low_ tumor	**0.0264**			NA	.123			NA
GZMA_high_/GZMA_low_ tumor	**0.0132**			NA	.106			NA
GZMB_high_/GZMB_low_ tumor	**0.0185**			NA	**.0034**			NA
PRF1_high_/PRF1_low_ tumor	**0.026**		.	NA	**.0017**			NA
CD8A_high_/CD8A_low_ tumor	**0.002**			NA	**< 0.0001**			NA
IFNG_high_/IFNG_low_ tumor	0.243			NA	.523			NA
TBX2_high_/TBX2_low_ tumor	**0.0182**			NA	.092			NA
TNF_high_/TNF_low_ tumor	**0.0245**	.217	.088-.536	**.001**	.089			NA
IATs_high_/IATs_low_ tumor	**< 0.0001**			NA	**< 0.0001**	.254	.105-.618	**.003**

Cox proportional hazards regression model; Variables associated with survival by univariate analysis were adopted as covariates in multivariate analyses.

OS, overall survival; DFS, disease-free survival; HR, hazard ratio; CI, confidence interval. NA, not applicable. Pos, positive. Neg, negative. The bold values indicate that the P < 0.05.

**Figure 5:**
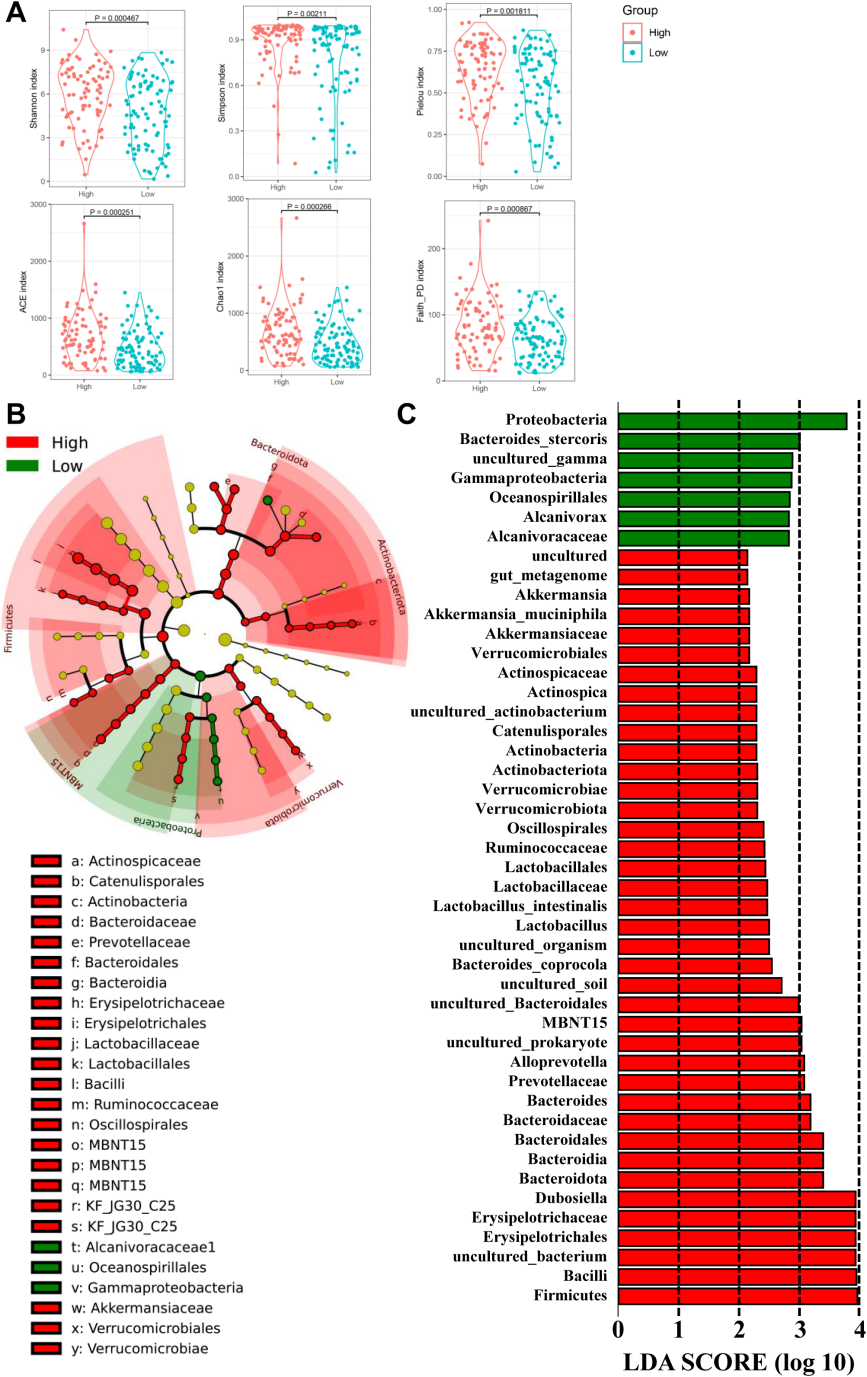
The diversity and richness of the gastric microbiota, and different bacterial taxa between the IATs^high^ group and IATs^low^ group. **(A)** The α-diversity indices (Shannon, Simpson, Pielou, ACE, Choa, and Faith_PD) evaluate the overall structure between the two groups, and **(B, C)** LEfSe identifies taxa with significant differences in abundance, shown if exceeding an LDA threshold value of >2.

To identify mucosal microbiota signatures associated with IATs expressing patterns, we grouped patients into IATs ^high^ group/IATs ^low^ group. Stratification revealed that the high IATs group had greater mucosal microbiota richness and diversity, as indicated by alpha diversity measures (**Figure 5A**). Discriminant analyses using LEfSe identified 46 bacterial phylotypes significantly different between IATs^high^ group and IATs^low^ group (**Figures 5B, C**), with specific enrichment of *Akkermansia_muciniphila*, *Lactobacillus_intestinalis*, *Bacteroides_coprocola*, *MBNT15*, *uncultured_prokaryote*, and other bacteria in the IATs^high^ group. We further uncovered an enrichment of specific taxa, including *Proteobacteria*, *Bacteroides_stercoris*, *uncultured_gamma*, *Gammaproteobacteria*, *Oceanospirillales*, *Alcanivorax*, and *Alcanivoracaceae* within IATs^low^ group. This suggests a potential link between mucosal microbiota composition and IATs expression, with implications for targeted therapeutic interventions.

### The association between discriminative species and distinctive selected IATs in GC mucosal tissues

**Figure 6:**
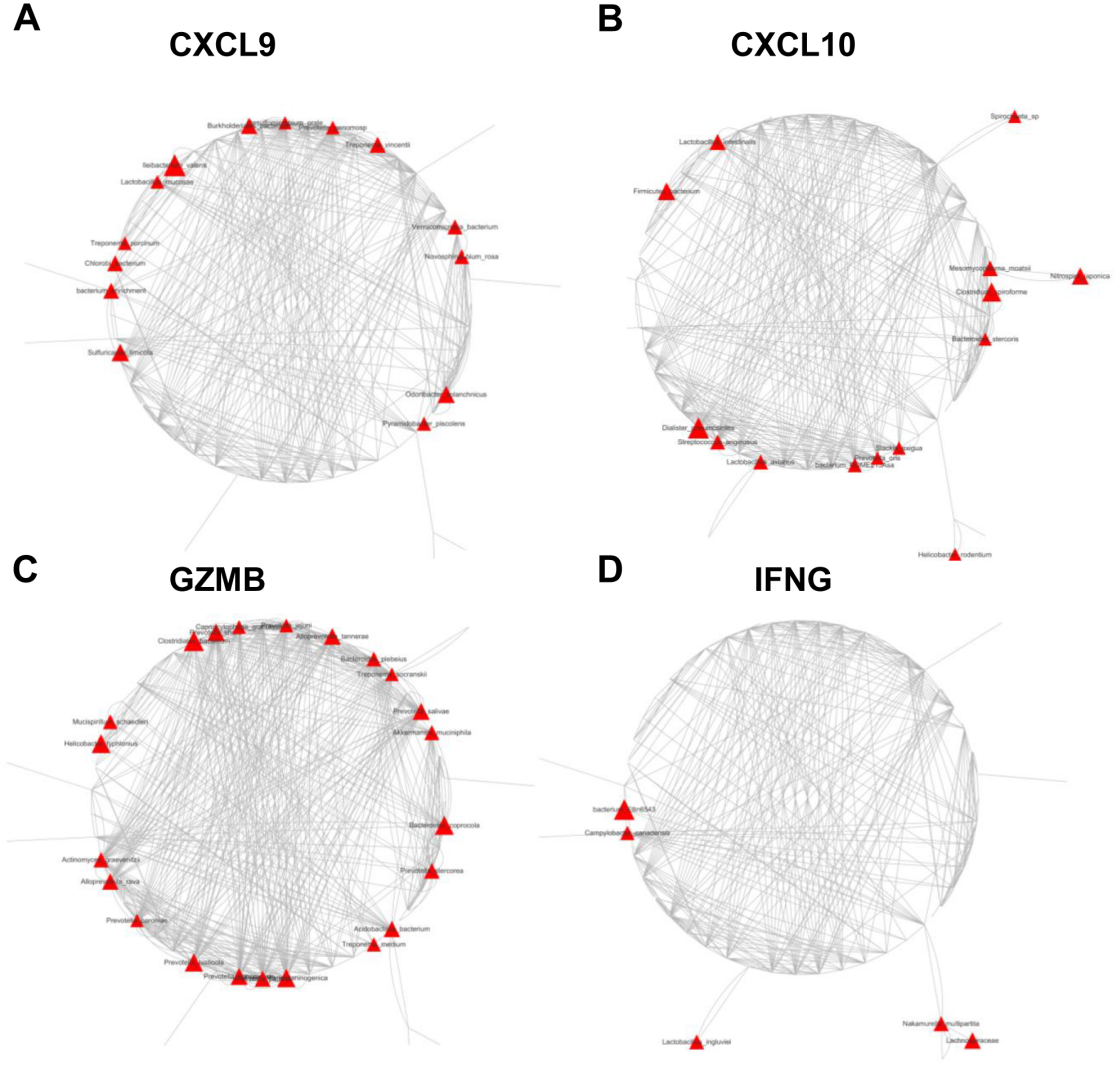
Network plots of operational taxonomic units (OTUs) based on mucosal abundance associated with each cytokine in the mucosal tissues of 85 GC patients. Nodes represent bacterial OTUs, and their abundance is significantly correlated with the expression of CXCL9 **(A)**, CXCL10 **(B)**, GZMB **(C)**, or IFNG **(D)**. The size of each node corresponds to the correlation coefficient.

To evaluate the effects of mucosal bacterial species on antitumor immune cell infiltration and the antitumor immune response, we used Spearman’s correlation analysis to assess the association between 1483 discriminative species and four distinctive IATs (CXCL9, CXCL10, GZMB and IFNG) in the GC tissues. This analysis showed that the expression of these chemokines and cytokines was significantly correlated with the abundance of several OTUs (**Figures 6A–D**). Notably, all corelated OTUs were positively corelated with these four chemokines. Specifically, for the chemokine CXCL9, the mucosal bacterial species showing significant positive correlations included *Burkholderiales_bacterium*, *Desulfomicrobium_orale*, *Prevotella_genomosp*, *Treponema_vincentii*, *Verrucomicrobia_bacterium*, *Novosphingobium_rosa*, *Odoribacter_splanchnicus*, *Pyramidobacter_piscolens*, *Sulfuricaulis_limicola*, *bacterium_enrichment*, *Chlorobi_bacterium*, *Treponema_porcinum*, *Lactobacillus_mucosae*, and *Ileibacterium_valen*. For the chemokine CXCL10, the significantly positively correlated mucosal bacterial species encompassed *Firmicutes_bacterium*, *Lactobacillus_intestinalis*, *Spirochaeta_sp*, *Mesomycoplasma_moatsii*, *Nitrospira_japonica*, *Clostridium:spiroforme*, *Bacteroides_stercoris*, *Helicobacter_rodentium*, *Slackia_exigua*, *Prevotella_oris*, *bacterium_ROME215Asa*, *Lactobacillus_aviarius*, *Streptococcus_anginosus*, and *Dialister_pneumosintes*. Regarding the cytokine GZMB, the significantly positively correlated mucosal bacterial species included *Alloprevotella_tannerae*, *Bacteroides_plebeius*, *Treponema_socranskii*, *Prevotella_salivae*, *Akkermansia_muciniphila*, *Bacteroides_coprocola*, *Prevotella_stercorea*, *Acidobacteria_bacterium*, *Treponema_medium*, *Prevotella_melaninogenica*, *Prevotella_nanceiensis*, *Prevotella_pallens*, *Prevotella_histicola*, *Prevotella_baroniae*, *Alloprevotella_rava*, *Actinomyces_graevenitzii*, *Helicobacter_typhlonius*, *Mucispirillum_schaedleri*, *Clostridiales_bacterium*, *Prevotella_shahii*, *Capnocytophaga_granulosa*, and *Pr*evotella_jejuni. Furthermore, with the cytokine IFNG, the significantly positively correlated mucosal bacterial species comprised *Nakamurella_multipartita*, *Lachnospiraceae*, *Lactobacillus_ingluviei*, *Campylobacter_canadensis*, and *bacterium_Ellin6543*. Finally, the bacterium *Akkermansia_muciniphila* may play a role in GZMB regulation, potentially influencing the tumor immune microenvironment. Its association with specific chemokine expression suggests its potential involvement in shaping the immune response within the tumor microenvironment, highlighting its significance in modulating the tumor’s immune landscape.

### Inferred functional changes in GC-associated gastric mucosal microbiota

**Figure 7:**
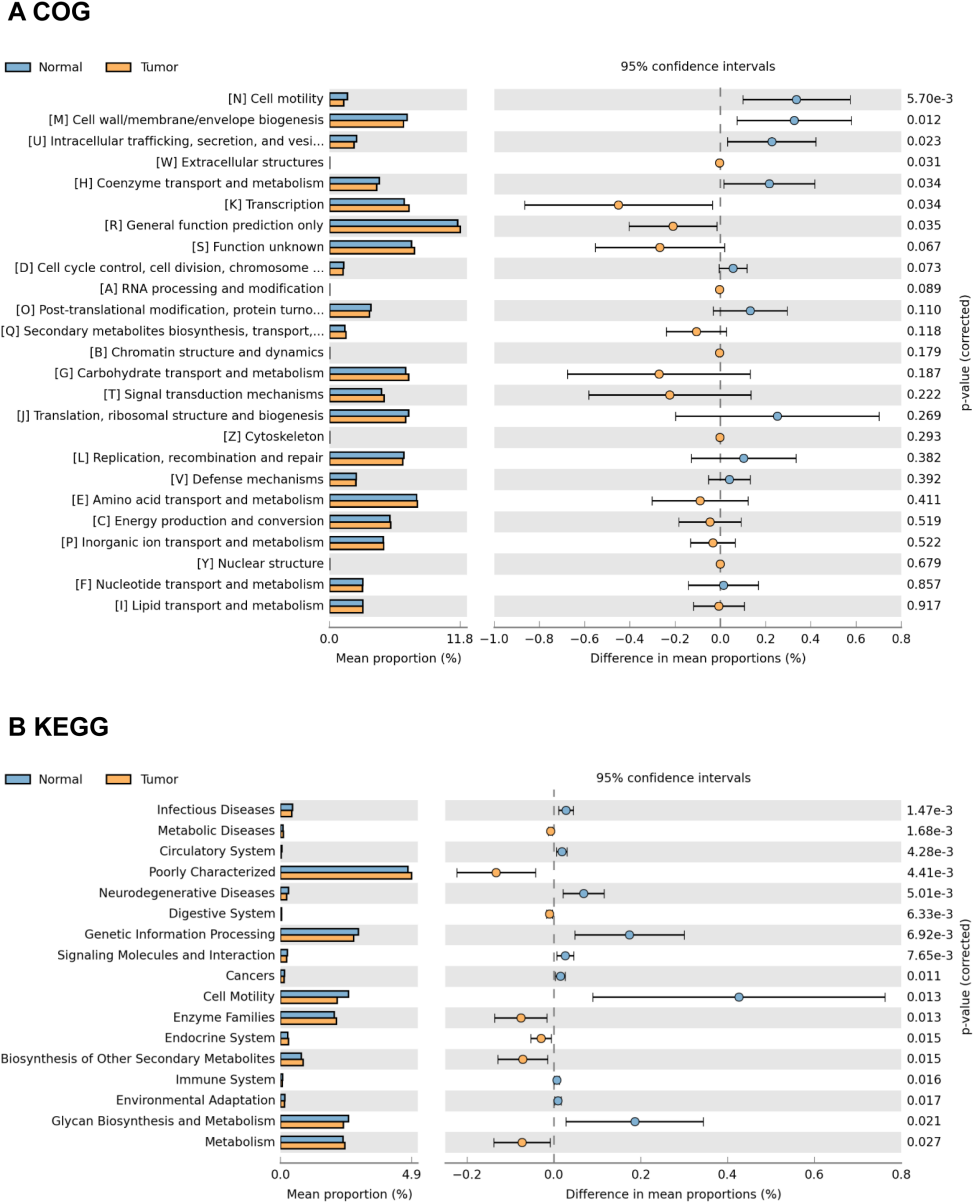
Representative COG functional category and KEGG functional pathways enriched in the tumor and adjacent normal mucosae microbiome. PiCRUSt-based analysis using Welch’s t-test reveals differences between the two groups, and multiple testing correction by the Benjamini-Hochberg method is applied based on the false discovery rate (FDR) by STAMP. Comparisons for each COG functional category **(A)** and KEGG functional pathway **(B)** are shown as percentages.

The functional content of the gastric microbiota was predicted by PiCRUSt based on closed-reference OTU picking. In our present study, 25 Clusters of Orthologous Groups (COG) functional categories were tested, identifying 7 differentially abundant COGs with a QFDR < 0.05 between the GC tumor and normal mucosae microbiota (**Figure 7A**). These 7 COG categories, including cell motility, cell wall/membrane/envelope biogenesis, intracellular trafficking, secretion, and vesicular transport, extracellular structures, coenzyme transport and metabolism, transcription and general function prediction only, exhibited the most significant differences between the GC tumor and normal mucosae microbiota. Among these differential COGs, extracellular structures, transcription and general function prediction only were significantly enriched in the tumoral microbiota. Additionally, we compared 40 Kyoto Encyclopedia of Genes and Genome (KEGG) pathways at level 2. At an FDR of 0.05, we identified 17 differentially abundant pathways between the GC tumor and normal mucosae microbiota (**Figure 7B**; **Supplementary Figure S5**). Consistent with the significant alterations in IATs-associated gastric microbiota, the KEGG pathways were changed between IATs^high^ group and IATs^low^ group in gastric mucosal tissues (**Supplementary Figure S6**). Together, these functional changes in the gastric microbiota may contribute to cytotoxic T cells infiltration and functional regulation.

## Discussion

In this study, our analysis of 16S rRNA gene sequencing data reveals a noteworthy increase in richness and evenness within GC tumor tissues compared to their non-tumor counterparts. The elevated alpha diversity metrics emphasize heightened microbial diversity in tumor tissues, aligning with findings from a previous study (24). This may be due to the decreased diversity caused by the dominance of *Helicobacter pylori* in non-tumor counterparts. The other way, diverse microbial communities in tumor tissues may include bacteria that produce metabolites capable of influencing tumor growth. Moreover, diverse microbial populations could influence the local immune microenvironment by modulating the immune-subsets which could support tumor growth by creating an immunosuppressive environment. However, Liu et al. observed decreased diversity and richness in peritumoral and tumoral tissues compared to non-tumor tissues (6). The inconsistent findings underscore the lack of consensus regarding the relationship between microbial diversity and gastric mucosal tissues.

The dominant phyla in the gastric microbiota include Firmicutes, Proteobacteria, Bacteroidota, Campilobacterota, Actinobacteria, Fusobacteriota, Verrucomicrobiota, Acidobacteriota, and Cyanobacteria. Remarkably, the relative abundance of Helicobacter is reduced in GC tumor tissues compared to non-tumor tissues, consistent with previous studies (20, 24). This decrease may be attributed to the loss of specialized glandular tissues and decreased acid secretion (25). Discriminant analyses reveal significant shifts in microbial taxa between tumor and non-tumor mucosae, exemplified by an increased Proteobacteria/Campylobacterales ratio in tumor mucosae, indicating altered microbial profiles associated with GC. Additionally, network analysis highlights the central role of oral bacteria (*Fusobacterium*, *Porphyromonas*, *Prevotella*, etc.) in both tumor and normal mucosae networks, emphasizing their profound influence on GC microbial ecology. Notably, the top 10 genera of the gastric microbiota, including *Lactobacillus* and *Streptococcus*, are identified. *Lactobacillus* may produce metabolites serving as an energy source for tumor growth and angiogenesis (26), while the abundance of *Streptococcus* is increased in GC tumor tissues (24, 27).

Our assessment of IATs in GC tumor tissues and matched non-tumor tissues revealed a correlation between heightened chemokine expression and increased microbiota richness and evenness in the IATs^high^ group. Consistent with previous studies (10, 28, 29), the majority of IATs are upregulated in tumor tissues, mirroring the trend of mucosal microbiota diversity. Microbial enrichments within IATs high expression tissues suggest potential therapeutic interventions targeting the tumor microbiota for improved clinical outcomes. Moreover, Spearman’s correlation analysis reveals significant associations between discriminative species and distinctive chemokines in GC tissues. Notably, the positive correlation of *Akkermansia_muciniphila* with specific chemokine expression underscores its potential role in modulating the GC tumor immune microenvironment. Recently studies have shown significant improvement in the pathological complete response rate among GC patients participating a randomized trial using perioperative PD-1 inhibitor plus chemotherapy for locally advanced GC (30). Studies have also demonstrated that intestinal microbiota composition significantly influences the effectiveness of anticancer immunosurveillance, impacting the therapeutic activity of immune-checkpoint inhibitors (ICIs) and immunogenic chemotherapies (31–33). Consistently, fecal microbiota transplantation (FMT) of microbiota from therapy-responding patients restored sensitivity to immunotherapy in germ-free environments or in mice treated with antibiotics and made insensitive to immunotherapy (34). Importantly, recent studies have further shown that response to ICIs therapy correlated with the percentage of *Akkermansia_muciniphila* in the intestinal microbiome of patients (35).

COG functional category tests showed that extracellular structures, transcription and general function prediction only were significantly enriched in the tumoral microbiota. Besides, KEGG enrichment analysis showed that pathways related to Amino acid metabolism including Isoflavonoid biosynthesis, Retinol metabolism, Lipoic acid metabolism, Adipocytokine signaling pathway, Fatty acid metabolism, and PPAR signaling pathway had significantly increased relative abundance in the tumoral microbiota. Liu et al. also reported that amino acid transport and metabolism exhibited the most significant differences among GC microhabitats (6). Studies have also shown that the PPAR signaling pathway is a crucial regulator in autocrine and paracrine signaling in the tumor microenvironment, modulating cancer-associated fibroblasts and tumor-associated macrophages/immune cells (36). Our observation of PPAR signaling pathway enrichment in GC tumor mucosal suggests pathway activation by the GC microbiome. Interestingly, we observed the enrichments of superpathway of L-lysine, L-threonine and L-methionine biosynthesis I, superpathway of arginine and polyamine biosynthesis, anhydromuropeptides recycling, superpathway of polyamine biosynthesis I, superpathway of L-methionine biosynthesis, reductive TCA cycle I, superpathway of S-adenosyl-L-methionine biosynthesis and tRNA processing in the IATs^high^ group mucosae microbiome. Increases in these pathways are predictive of bacterial involvement in amino acid metabolism by the gut microbiome, which has been linked to hyperproliferation of cells in the tumor microenvironment (37, 38). The enrichment of these pathway in IATs^high^ group mucosae microbiome highlights their potential contribution to the immune response. Furthermore, pathways involved in polyamine biosynthesis have been reported to remodel the tumor immune microenvironment by altering the activation and proliferation of CD4^+^ and CD8^+^ T lymphocytes (39, 40). The association of polyamine biosynthesis with IATs in this study supports the role that this pathway may play in CD8^+^ T lymphocytes remodeling and supports previous observations in GC (41). Further investigations into the implications of microbiome functional dysbiosis in IATs^high^ and IATs^low^ groups are needed for a deeper understanding of the gastric immune microenvironment.

Our study had several limitations. First, the sample size is relatively small, resulting in the lack of significant correlation between clinical features and microbiome, and between clinical features and metabolome. Second, we did not perform longitudinal studies since we could not obtain serial tissue samples from the recruited patients. Third, we did not include gastric cancer patients from different regions, so our patient heterogeneity is insufficient. Fourth, the diet could heavily influence both the gastric microbiota and metabolites, but we could not obtain the diet information of patients to analyze the effect of diet on gastric microbiome and metabolome.

In conclusion, our study provides insights into the microbiome of GC tumor tissues and matched non-tumor tissues, unveiling IATs-associated bacteria and highlighting the pivotal role of mucosal microbiota alterations in GC. The identification of potential biomarkers and therapeutic targets, such as IATs-associated bacteria, offers prospects for improving clinical outcomes in GC. Further research is warranted to delve into the functional implications of microbiome dysbiosis in IATs^high^ and IATs^low^ group mucosae, advancing our understanding of the gastric immune microenvironment.

## Materials and methods

### Patients and database

A total of 85 individuals, scheduled for primary tumor resection at the Affiliated Hospital of Jiangnan University between 2016 and 2019, were enrolled in the study. Exclusion criteria included prior chemo-radiotherapy. Patients received no antibiotics within a month before surgery but were administered intravenous antibiotics shortly before resection. Post-surgery, 85 paired fresh tissues, including gastric tumor and matched non-tumor tissues, were collected. Biopsies were snap-frozen in cryovial immediately with liquid nitrogen and then stored at -80°C until DNA extraction. Histopathological and clinical findings were scored according to the International Union Against Cancer (UICC)-TNM staging system.

### DNA extraction and16S rRNA gene sequencing

A total of 170 tissue samples (one tumor and one adjacent normal sample per individual) were processed for DNA purification. The DNA extraction was carried out according to the AllPrep DNA/RNA extraction kit and total RNA were extracted using the Ultrapure RNA Kit (CWBIO, China). Total DNA was purified from tumor and paired normal adjacent mucosal tissue samples. Mucosa-associated microbiota was analyzed through 16S rRNA sequencing. 16S rRNA gene amplicon sequencing was carried out employing the 16S Meta-genomic Sequencing Library Preparation protocol developed by Illumina (San Diego, California, USA. Briefly, 200 ng of mucosal DNA was amplified from each sample using the primers 515F (5′ GTGCCAGCMGCCGCGGTAA 3′) with Titanium Adaptor B and 806R (5′ GGACTACHVGGGTWTCTAAT 3′) with Titanium Adaptor A and a sample‐specific barcode sequence consisting of twelve nucleotides targeting the V4 hypervariable region of the 16S rRNA gene using FastStart Taq DNA Polymerase (Roche). The resulting sequences were processed for bioinformatics analysis.

### RNA isolation, chemokine mRNA expression and quantitative PCR

Total RNA from GC tumor and paired normal adjacent tissues was isolated using Trizol (Invitrogen, USA). cDNA was synthesized using Superscript III Reverse Transcriptase (Promega, USA). Real-time PCR reactions were conducted with SYBR Green (TaKaRa, Japan) and analyzed on the Step One Plus Real-time PCR System (Applied Biosystems, USA) with the following conditions: 95°C for 5 min, 95°C for 5 s, 60°C for 30 s, for 40 cycles. The relative mRNA expression value was calculated by 2 -△△T method. GAPDH was utilized as the internal control. The primers used were as follows: CXCL9, forward primer F(5′ AAGCAGCCAAGTCGGTTAGT 3′) and reverse primer R(5′ CAGCAGTGTGAGCAGTGATTC 3′); CXCL10, forward primer F(5′ AGCAGAGGAACCTCCAGTCT 3′) and reverse primer R(5′ AGGTACTCCTTGAATGCCACT 3′); GZMA, forward primer F(5′ GAAGAGACTCGTGCAATGGAGA 3′) and reverse primer R(5′ AAGGCCAAAGGAAGTGACCC 3′); GZMB, F(5′ CCAGGGCAGATGCAGACTTT 3′) and reverse primer R(5′ CTCGTATCAGGAAGCCACCG 3′); PRF1, F(5′ GGGGCTGATGCCACCATT 3′) and reverse primer R(5′ GGCACTTGGGCTCTGGAAT 3′); CD8A, F(5′ CGGTTTCCTGGGGTAACAGT 3′) and reverse primer R(5′ TGCCTGAATCAGCCTTTCTGT 3′); IFNG, F(5′ GAGTGTGGAGACCATCAAGGA 3′) and reverse primer R(5′ TGGACATTCAAGTCAGTTACCGAA 3′); TBX2, F(5′ TACGAGGAGCACTGCAAACC 3′) and reverse primer R(5′ CACGACTTCTCCTCAGCTCG 3′); TNF, F(5′ AGCCCATGTTGTAGCAAACC 3′) and reverse primer R(5′ ATGAGGTACAGGCCCTCTGA 3′); GAPDH, forward primer F(5′ TGACTTCAACAGCGACACCCA 3′) and reverse primer R(5′ CACCCTGTTGCTGTAGCCAAA 3′). Experiments were performed in triplicate.

### Bioinformatics analysis

Microbiome bioinformatics were performed using QIIME 2 (2023.9) with slight modification according to the official tutorials. Briefly, raw sequence data were demultiplexed using the demux plugin following by primers cutting with cutadapt plugin (Martin, M., 2011). Sequences were then quality filtered, denoised, merged and chimera removed using the DADA2 plugin. Species annotation was performed using QIIME2 software. The annotation database is Silva Database. Alpha and beta diversity analyses were calculated with QIIME2 and displayed with R software (Version 3.6.2). Principal Coordinate Analysis (PCoA) was carried out to obtain principal coordinates and visualize differences of samples in complex multi-dimensional data. A matrix of weighted or unweighted unifrac distances among samples obtained previously was transformed into a new set of orthogonal axes, where the maximum variation factor was demonstrated by the first principal coordinate, and the second maximum variation factor was demonstrated by the second principal coordinate, and so on. The three-dimensional PCoA results were displayed using QIIME2 package, while the two-dimensional PCoA results were displayed using ade package and ggplot2 package in R software (Version 3.6.2).

## Data Availability

The datasets presented in this study can be found in online repositories. The names of the repository/repositories and accession number(s) can be found below: NCBI SRA database under accession number PRJNA1032279.
