# Supplementary material for: Mucosal microbiota characterization in gastric cancer identifies immune-activated–related transcripts relevant gastric microbiome signatures
Source: Front Immunol. 2024 Sep 23;15:1435334. doi: 10.3389/fimmu.2024.1435334 (PMC11456469; doi:10.3389/fimmu.2024.1435334)
Supplement: Supplementary file 1 [file DataSheet1.pdf]

**Figure S1.**

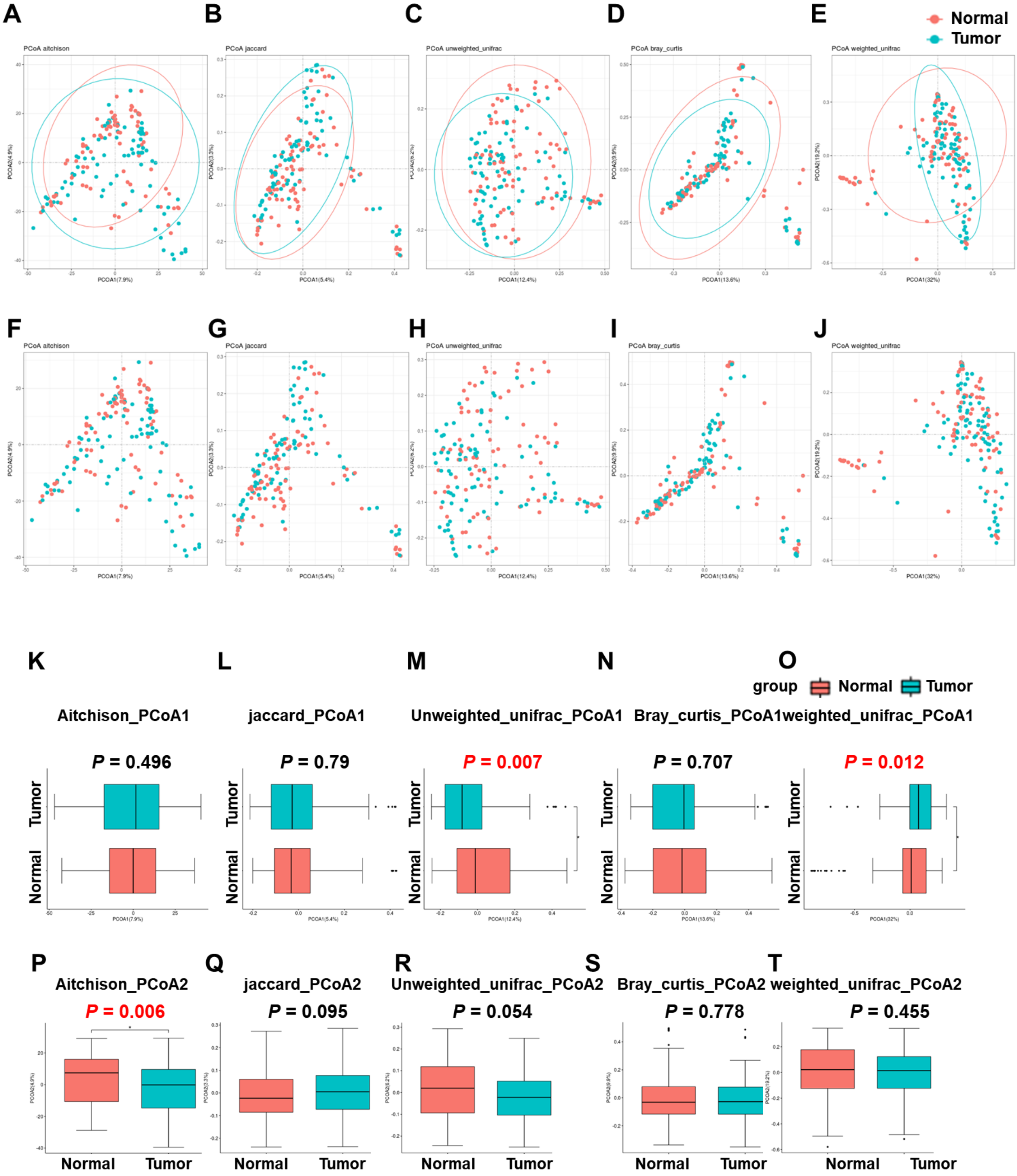

A
B
C
D
E
Normal
Tumor
PCoA aitchison
PCoA jaccard
PCoA unweighted_unifrac
PCoA bray_curtis
PCoA weighted_unifrac
F
G
H
I
J
K
L
M
N
O
group Normal Tumor
Aitchison_PCoA1
jaccard_PCoA1
Unweighted_unifrac_PCoA1
Bray_curtis_PCoA1
weighted_unifrac_PCoA1
P = 0.496
P = 0.79
P = 0.007
P = 0.707
P = 0.012
P
Q
R
S
T
Aitchison_PCoA2
jaccard_PCoA2
Unweighted_unifrac_PCoA2
Bray_curtis_PCoA2
weighted_unifrac_PCoA2
P = 0.006
P = 0.095
P = 0.054
P = 0.778
P = 0.455
Normal
Tumor

**Figure S2.**

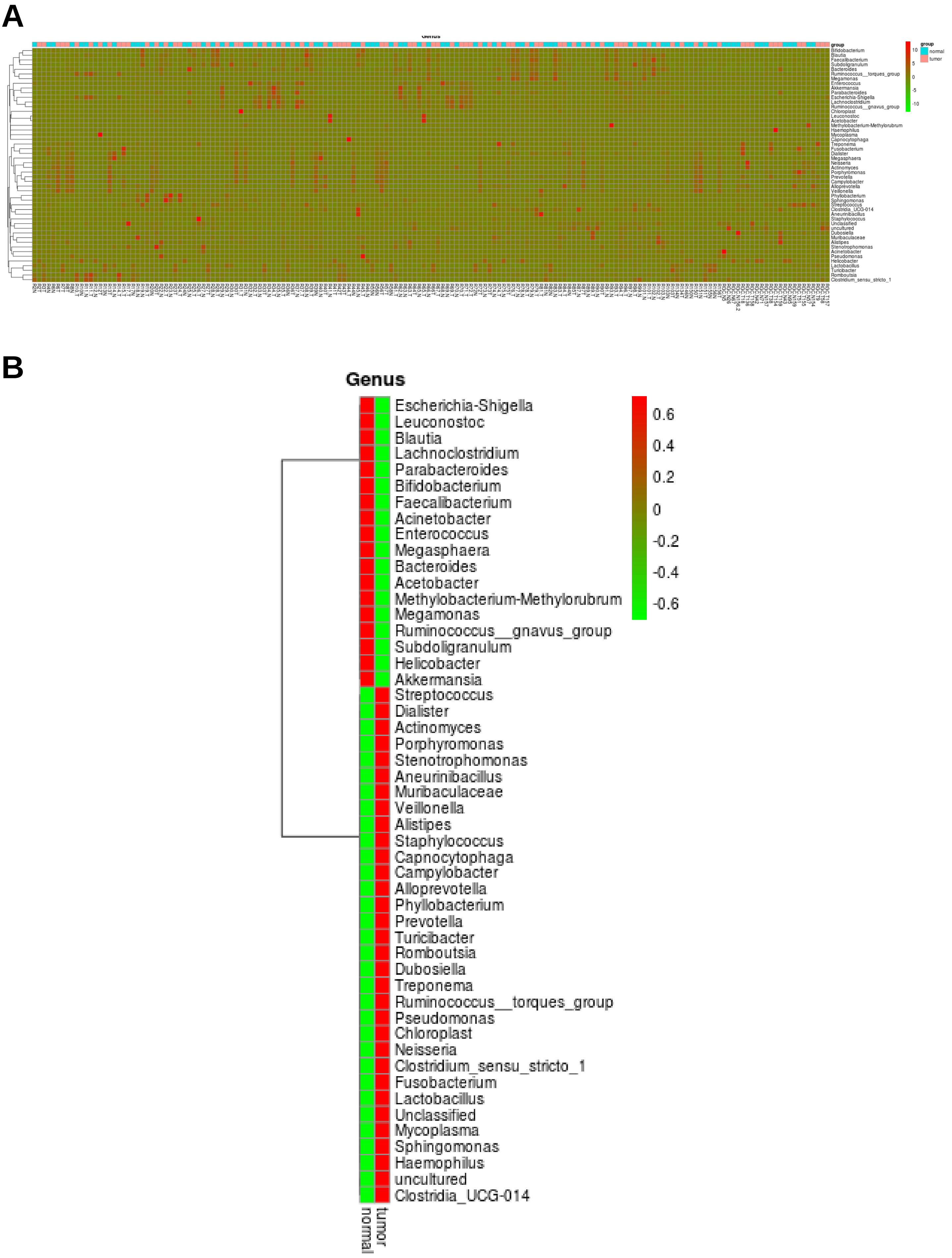
A
B
Genus
Escherichia-Shigella
Leuconostoc
Blautia
Lachnoclostridium
Parabacteroides
Bifidobacterium
Faecalibacterium
Acinetobacter
Enterococcus
Megasphaera
Bacteroides
Acetobacter
Methylobacterium-Methylorubrum
Megamonas
Ruminococcus__gnavus_group
Subdoligranulum
Helicobacter
Akkermansia
Streptococcus
Dialister
Actinomyces
Porphyromonas
Stenotrophomonas
Aneurinibacillus
Muribaculaceae
Veillonella
Alistipes
Staphylococcus
Capnocytophaga
Campylobacter
Alloprevotella
Phyllobacterium
Prevotella
Turicibacter
Romboutsia
Dubosiella
Treponema
Ruminococcus__torques_group
Pseudomonas
Chloroplast
Neisseria
Clostridium_sensu_stricto_1
Fusobacterium
Lactobacillus
Unclassified
Mycoplasma
Sphingomonas
Haemophilus
uncultured
Clostridia_UCG-014
0.6
0.4
0.2
0
-0.2
-0.4
-0.6
normal
tumor

**Figure S3.**

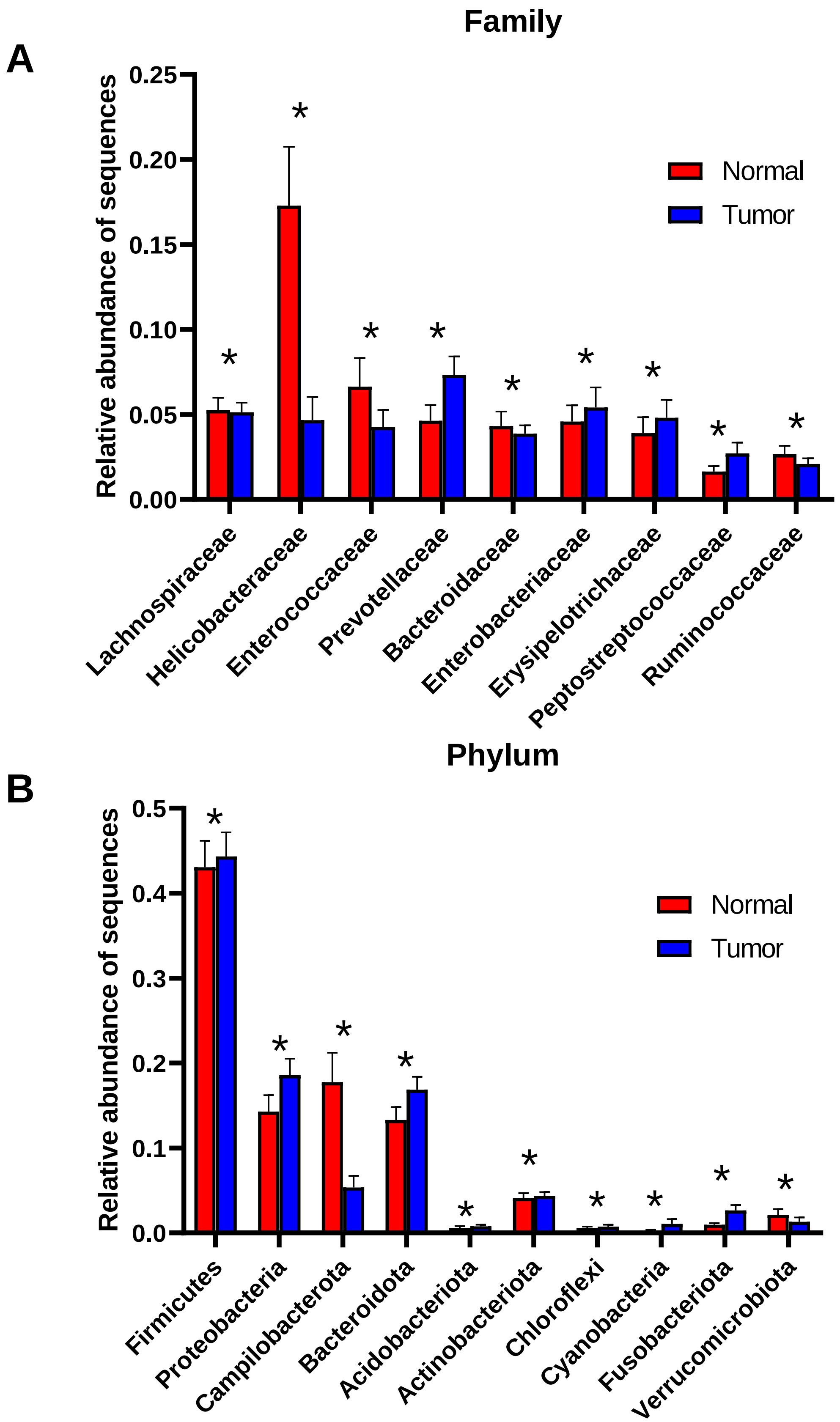


**Figure S3. Different bacterial taxa between tumor and matched normal mucosae microbiomes.** Comparisons of the relative abundance of dominant bacterial taxa at the level of bacterial family (A) and phylum (B).

# Figure S4

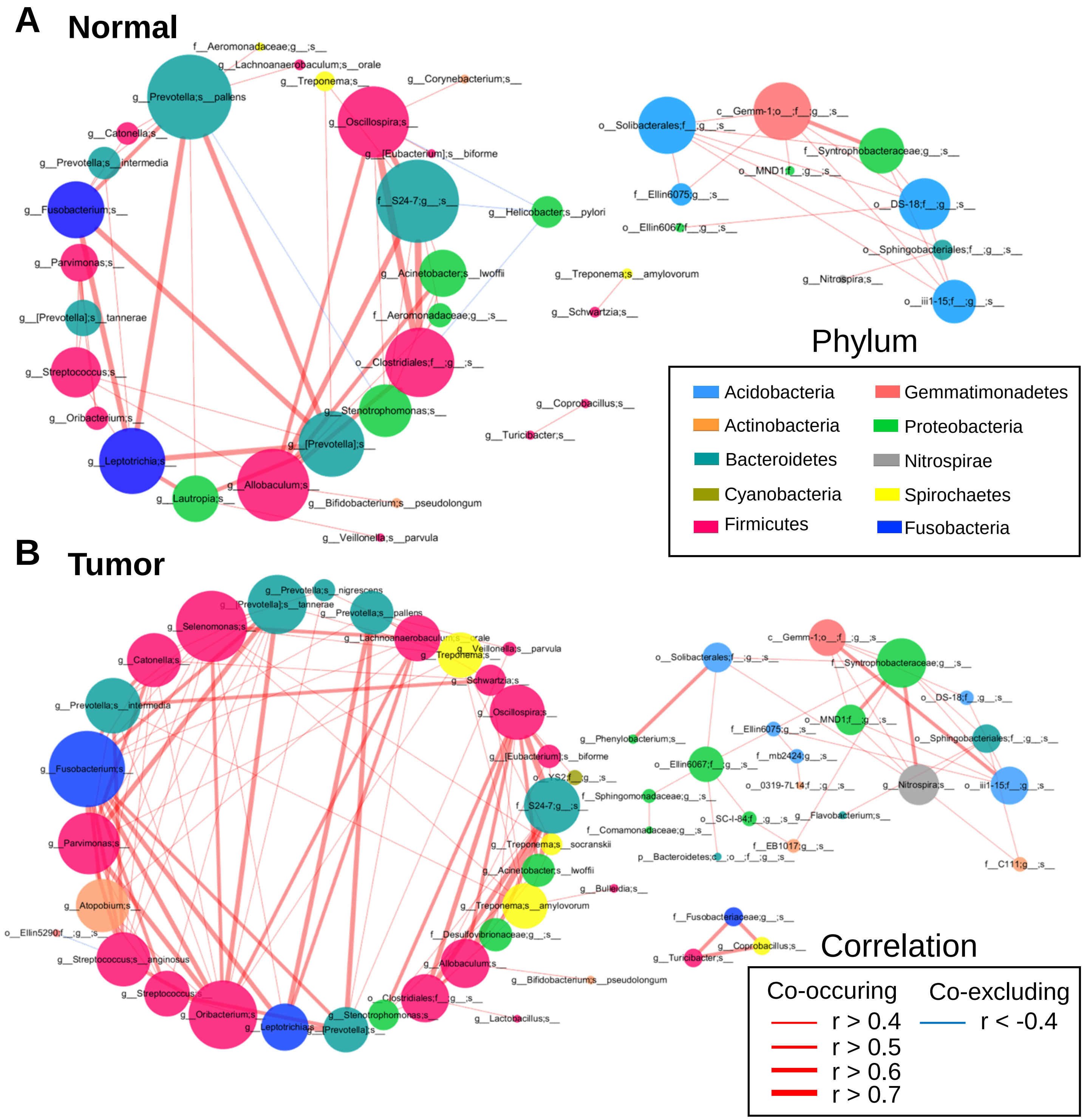


**Figure S4. Correlation network of the abundant gastric microbiota in normal mucosae** (A), **matched tumor mucosae** (B). The correlation coefficients were calculated with the Sparse Correlations for Compositional data algorithm (SparCC). Cytoscape version 3.7.0 was used for network construction. Red and blue lines represent positive and negative correlations, respectively.

# Figure S5

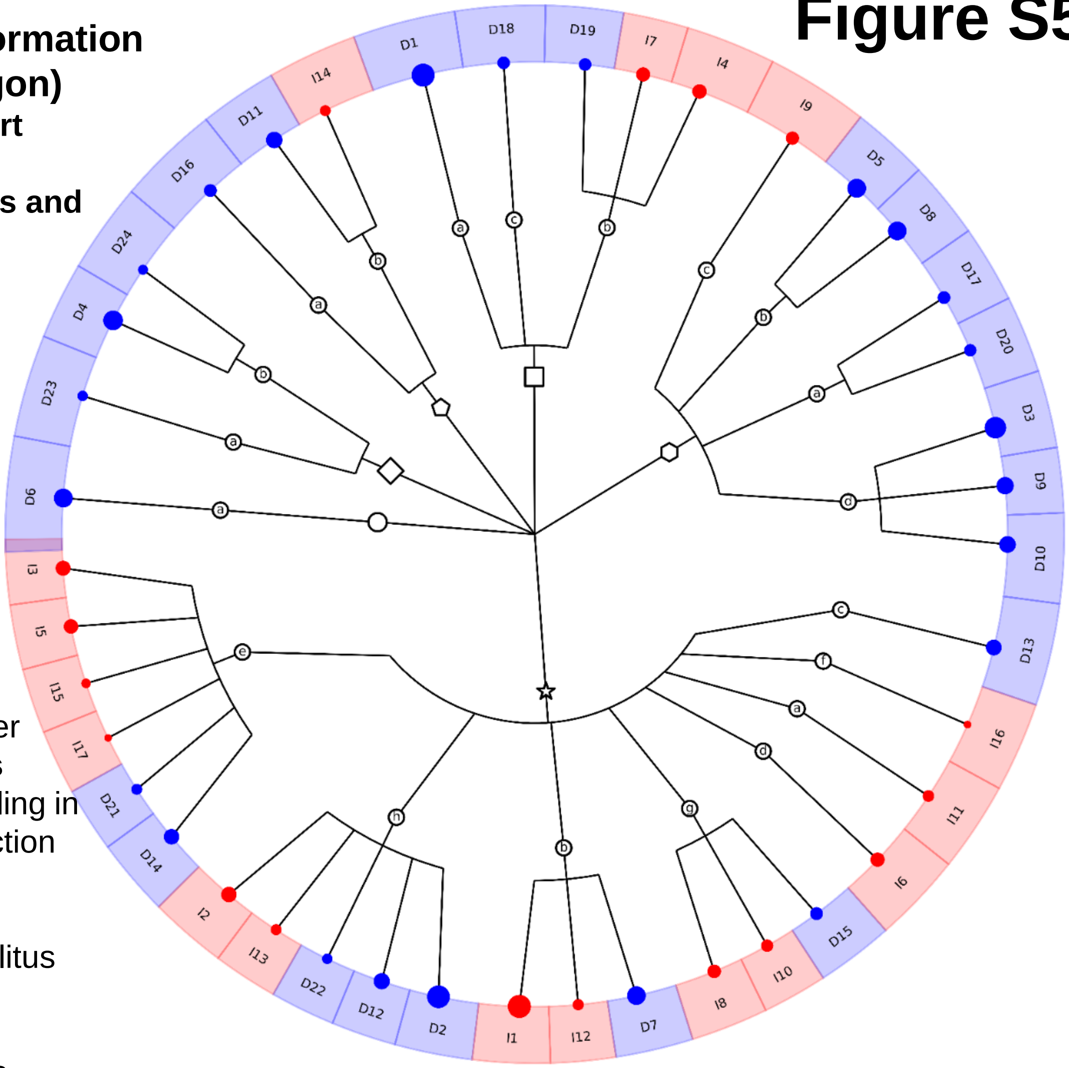


## Environmental Information Processing(pentagon)

**a: Membrane Transport**
**D16:** Secretion system
**b: Signaling Molecules and Interaction**
**D11:** Bacterial toxins
**I14:** Cellular antigens

## Cellular Processes(circle)

**a: Cell Motility**
**D6:** Flagellar assembly

## Human Diseases (hexagon)

**a: Cancers**
**D17:** Prostate cancer
**D20:** Pathways in cancer
**b: Infectious Diseases**
**D5:** Epithelial cell signaling in Helicobacter pylori infection
**D8:** Amoebiasis
**c: Metabolic Diseases**
**I9:** Type II diabetes mellitus
**d: Neurodegenerative Diseases**
**D3:** Parkinson's disease
**D9:** Alzheimer's disease
**D10:** Huntington's disease

## Organismal Systems (square)

**a: Circulatory System**
**D1:** Cardiac muscle contraction
**b: Endocrine System**
**D19:** Progesterone-mediated oocyte maturation
**I7:** PPAR signaling pathway
**I4:** Adipocytokine signaling pathway
**c: Immune System**
**D18:** Antigen processing and presentation

## Genetic Information Processing(diamond)

**a: Folding, Sorting and Degradation**
**D23:** Protein folding and associated processing
**b: Replication and Repair**
**D4:** Restriction enzyme
**D24:** Replication, recombination and repair proteins

## Metabolism(star)

**a: Amino acid metabolism**
**I11:** Amino acid metabolism
**b: Biosynthesis of Other Secondary Metabolites**
**D7:** Biosynthesis and biodegradation of secondary metabolites
**I12:** Streptomycin biosynthesis
**I1:** Isoflavonoid biosynthesis
**c: Glycan Biosynthesis and Metabolism**
**D13:** Lipopolysaccharide biosynthesis proteins
**d: Lipid Metabolism**
**I6:** Fatty acid metabolism
**g: Metabolism of Terpenoids and Polyketides**
**D15:** Tetracycline biosynthesis
**I10:** Polyketide sugar unit biosynthesis
**I8:** Biosynthesis of vancomycin group antibiotics
**e: Metabolism of Cofactors and Vitamins**
**D14:** Biotin metabolism
**D21:** Folate biosynthesis
**I17:** Nicotinate and nicotinamide metabolism
**I15:** One carbon pool by folate
**I5:** Lipoic acid metabolism
**I3:** Retinol metabolism
**f: Metabolism of Other Amino Acids**
**I16:** Selenocompound metabolism
**h: Xenobiotics Biodegradation and Metabolism**
**D2:** Atrazine degradation
**D12:** Nitrotoluene degradation
**D22:** Polycyclic aromatic hydrocarbon degradation
**I13:** Drug metabolism - other enzymes
**I2:** Ethylbenzene degradation

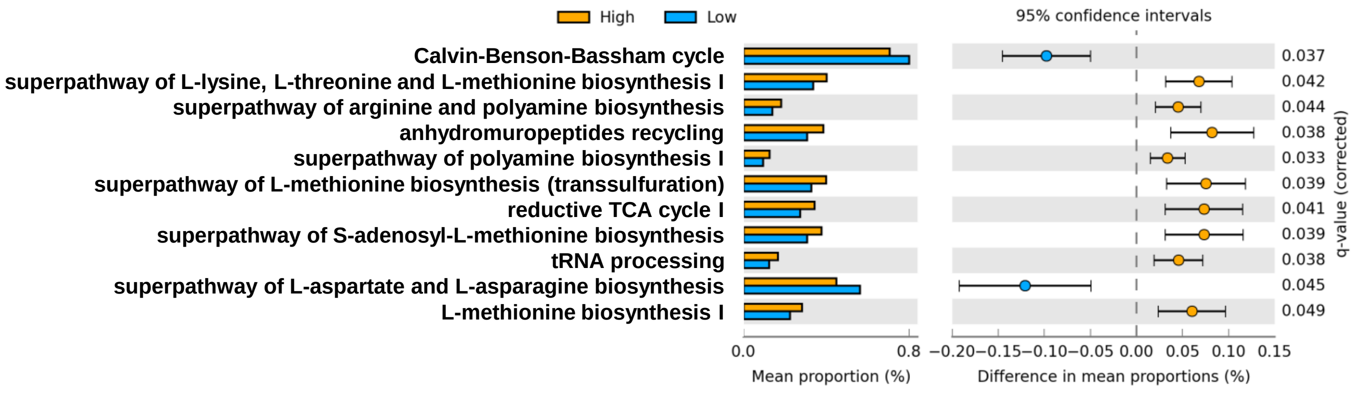


**Figure S6. Representative KEGG functional pathways that are enriched in the IATs$^{high}$ group mucosae microbiome.**

PiCRUSt-based gastric mucosae microbiome study in the IATs$^{high}$ group and IATs$^{low}$ group. The different bacterial functions between the two groups were evaluated based on two-sided Welch's t-test . The Benjamini-Hochberg method was used for multiple testing correction based on the false discovery rate (FDR) by STAMP. Comparisons between the IATs$^{high}$ group and IATs$^{low}$ group mucosae microbiome for each KEGG functional pathways shown by percentage.
